# Expression of three GnRH receptors in specific tissues in male and female sea lampreys *Petromyzon marinus* at three distinct life stages

**DOI:** 10.3389/fnins.2013.00088

**Published:** 2013-05-28

**Authors:** Jeffrey A. Hall, Wayne A. Decatur, Dana M. Daukss, Mary K. Hayes, Timothy J. Marquis, Scott J. Morin, Thomas F. Kelleher, Stacia A. Sower

**Affiliations:** Department of Molecular, Cellular and Biomedical Sciences, Center for Molecular and Comparative Endocrinology, University of New HampshireDurham, NH, USA

**Keywords:** gonadotropin-releasing hormone receptors, lamprey, basal vertebrate, receptor, hormone, pituitary

## Abstract

Two recently cloned gonadotropin-releasing hormone (GnRH) receptors (lamprey GnRH-R-2 and lamprey GnRH-R-3) along with lamprey (l) GnRH-R-1 were shown to share similar structural features and amino acid motifs common to other vertebrate receptors. Here we report on our findings of RNA expression of these three GnRH receptors in the three major life stages (larval, parasitic, and adult phases) of the sea lamprey, *Petromyzon marinus*, a basal vertebrate. For each stage, we examined the expression of messenger RNA encoding the receptors in the brain, pituitary, gonad, heart, muscle, liver, eye, intestine, kidney, skin, thyroid, gill, and endostyle by RT-PCR. In adult lampreys, the spatial expression of the three receptors in the brain and pituitary was investigated by *in situ* hybridization. In general, the receptors were more widely expressed in adult tissues as compared to parasitic-phase tissues and least widely expressed in the larval tissues. There were noted differences in male and female lampreys in the adult and parasitic phases for all three receptors. The data showed the presence of all three receptor transcripts in brain tissues for adult and parasitic phases and all three receptor transcripts were expressed in the adult pituitaries, but not in the parasitic pituitaries. However, in the larval phase, only lGnRH-R-1 was expressed in the larval brain and pituitary. *In situ* hybridization revealed that lGnRH-R-2 and -3 were expressed in the pineal tissue of adult female lampreys while lGnRH-R-1 was expressed in the pineal in adult male lampreys, all restricted to the pineal pellucida. In summary, these data provide an initial comparative analysis of expression of three lamprey GnRH receptors suggesting differential regulation within males and females at three different life/reproductive stages.

## Introduction

Lampreys, representative of only two extant agnathans, are the earliest evolved vertebrates for which there is a demonstrated neuroendocrine system. Lampreys have three functional hypothalamic gonadotropin-releasing hormone (GnRHs) (lGnRH-I, -II, and -III) and three functional pituitary GnRH receptors (lGnRH-R-1, -2 and -3) likely involved in mediating reproductive processes (Sower et al., 2009; Joseph et al., 2012). We currently have classified the lamprey GnRH receptors as follows: lGnRH-R-2 and -3 are Type III receptors and lGnRH-R-1 is considered an ancestral form that is more closely related to the type II GnRH-Rs (Joseph et al., 2012; Sower et al., 2012). In our recent study in which the two novel forms of GnRH receptors were cloned, lGnRH-R-2 precursor transcript was detected in a wide variety of tissues including the pituitary, whereas lGnRH-R-3 precursor transcript was not as widely expressed, primarily in the brain and eyes of adult male and female lampreys (Joseph et al., 2012). However, these studies did not examine the spatial relations or localization of these receptors in the brain of the lampreys nor examine the distribution in the three major life stages of the sea lamprey. Therefore, our objective was to examine expression of messenger RNA encoding the receptors in the brain, pituitary, gonad, heart, muscle, liver, eye, intestine, kidney, skin, thyroid, gill, and endostyle by RT-PCR in sea lampreys, *Petromyzon marinus*, at three life stages. In adult lampreys, the spatial expression of the three receptors in the brain and pituitary was investigated by *in situ* hybridization to gain an understanding of potential physiological significance.

Attribution of physiological significance to each receptor type by investigating the spatial expression of GnRH receptors is further complicated as studies have shown that more than one receptor type can be expressed in the same tissue. For example, in the zebrafish, the anatomical distribution of the four GnRH receptors (type III and type I) is widespread in the brain, eyes and gonads and additionally, all four of the GnRH receptors are expressed in the pituitary (Tello et al., 2008). The European sea bass possesses five isoforms of GnRH receptors (type III and type I) and all but one are expressed in the pituitary (Moncaut et al., 2005). In goldfish, two subtypes of the GnRH receptor have been identified (both type I) (Illing et al., 1999) and they are both expressed in the pituitary. Our initial expression studies on the GnRH receptors in lampreys indicate that, like other species, there are specific receptor subtypes within different tissues. The current studies were undertaken to determine if there was a predominance of one receptor subtype compared with the others in a particular tissue in the sea lamprey.

The sea lamprey has a unique life style and is the only vertebrate to have a parasitic phase, therefore we wanted to examine the expression of the three receptors in each of the three major life stages. There are approximately 40 species of lampreys that are classified as parasitic or non-parasitic. Lampreys spawn only once in their lifetime, after which they die, thus sexual maturation is a synchronized process coordinated with the three major life stages of the lamprey (larval, immature gonads; parasitic, maturing gonads; and adult, final maturation) (Sower, 2003). The sea lampreys begin their lives in freshwater as blind, filter-feeding larvae. After approximately 5–7 years in freshwater streams, metamorphosis occurs and the ammocoetes become free-swimming, sexually immature lampreys, which migrate to the sea or lakes for an approximately 15 month-long parasitic sea phase. After this period at sea, lampreys return in late spring to freshwater streams and undergo the final maturational processes resulting in mature eggs and sperm, spawning, and then death. Here we report on our findings of RNA expression for each of the three GnRH receptors in the three major life stages (larval, parasitic, and adult phases) of the sea lamprey.

## Materials and methods

### Collection and sampling

Adult sea lampreys, averaging 900 g each, were collected from the fish ladder on the Cocheco River in Dover, New Hampshire in late May and early June over two successive seasons (2011, 2012) during the lampreys' upstream spawning migration from the ocean to coastal rivers. The lampreys were transported to the Anadromous Fish and Aquatic Invertebrate Research (AFAIR) Laboratory, a freshwater fish facility at the University of New Hampshire (UNH), and maintained in an artificial spawning channel supplied with flow-through water from a nearby stream-fed reservoir at an ambient temperature range of 13–20°C, under natural photoperiod. Parasitic tissues were collected from control animals of landlocked parasitic lampreys, about 75 g each, adapted to seawater and fed on Atlantic cod (Gadus morhua) as described in Wong et al. (2012). Larval lampreys, approximately 2 g each, were collected in rivers connected to Lake Huron in Michigan and transported to UNH by Hammond Bay Biological Station (Millersburg, MI). Upon arrival, the larval lampreys were immediately sampled for tissues. All procedures for animal use followed the UNH Institutional Animal Care and Use guidelines.

### RNA isolation

Total RNAs were isolated from the brain, pituitary, gonads, heart, muscle, liver, eye, intestine, kidney, skin, thyroid, and gill tissues [tissues that were examined for GnRH receptors in previous studies, Silver et al. (2005), Joseph et al. (2012)]. Samples were collected from 20 larval lampreys, three male and three female parasitic lampreys and four male and four female adults. Following decapitation, individual tissues were dissected, snap frozen in liquid nitrogen and stored at −80°C. Total RNA was isolated from 100 mg of each tissue from each animal using QIAzol Lysis Reagent (QIAGEN, Valencia, CA) as described by Daukss et al. (2012) except for the pituitary (adenohypophysis) tissues. The concentration and purity of the total RNA was determined by UV spectroscopy at 260 nm and 280 nm using a NanoDrop 2000c spectrophotometer (ThermoScienitifc, Wilmington, DE) and then adjusted to 200 ng/μl each. Next equal amounts (20 μl) of that tissue's RNA from each animal were pooled and then stored at −80°C ready for cDNA synthesis. The pituitaries (total mass less than 100 mg/pool) were pooled into one tube for extraction. Similarly, because of their small size the larval tissues were also pooled into one tube for extraction.

### cDNA synthesis

First strand cDNA was synthesized in a 10 μl final reaction volume containing 1 μg of the total RNA, 100 U of Superscript III reverse transcriptase (Invitrogen, Carlsbad, California) and 200 ng of NotI-(dT)18 primer according to Daukss et al. (2012). These cDNA syntheses were tested by PCR amplification of Elongation Factor 1α (Joseph et al., 2012).

### RT-PCR

Several gene specific primers were selected against the cDNA sequence of the three GnRH receptors using PrimerSelect (Lasergene 8.0.2, DNASTAR, Madison, WI) and were obtained from Integrated DNA technology (Coralville, IA). Primer pairs were selected based on the length of amplicon, the number of exon/intron boundaries crossed and the reaction success when paired together. This resulted in the following: for R-1, lGnRHrGSP5 (forward) and lGnRHrGSP23 (reverse); for R-2, JAH 018 (forward) and lGnRHR2 Rev3 (reverse); for R-3, JAH 011 (forward) and JAH 016 (reverse) (Table 1).

**Table 1:** **Sequences of the primers used in the various PCR and RNA probe synthesis reactions**.

lGnRHrGSP5 (R-1, forward):	5′-CCGAACGCCAGCCACACAGGC-3′
lGnRHrGSP23 (R-1, reverse):	5′-CCACCACTGGCATCACAGAACG-3′
JAH 018 (R-2, forward):	5′-ATCGCCCACGTGGACCGTCACTTGGCAGAT-3′
lGnRHR2 Rev3 (R-2 reverse):	5′-GTGCTTGTGGTTGTGAAT-3′
JAH 011 (R-3, forward):	5′-CCAGCCGGGCCCGCAGTTCAC-3′
JAH 016 (R-3, reverse):	5′-CTGCAGCGGGGCGGAGAGGTC-3′
lGSP13 (R-1, forward):	5′-GCCCCTCCGAACGCCAGCCACACA-3′
lGSP8 (R-1, reverse):	5′-CGGCAGACGAACTCGCCCGC-3′
TKF1R2 (R-2, forward):	5′-ATGAATTCATCATCGCCCAC-3′
ABRev1bR2 (R-2 reverse):	5′-AAGCTGGTGGCCATGTACTC-3′
TKF1R3 (R-3, forward):	5′-ATGACGTTACTAGCGCACGC-3′
TK2R4R3 (R-3, reverse):	5′-CTCTTCACCCTCCTCGTGAT-3′

RT-PCR reactions were performed using a Mastercycler pro thermal cycler (Eppendorf, Hamburg, Germany) under the following conditions: 95°C/2 min; (95°C/30 s; 70°C/2 min 30 s) ×5 cycles; (95°C/30 s; 65°C/1 min; 68°C/1 min) ×5 cycles; (95°C/30 s; 60°C/1 min; 68°C/1 min) ×30 cycles; 68°C/10 min; 4°C/hold. The reaction products were analyzed by electrophoresis. Approximately 20 ng of cDNA was used in the corresponding PCR reactions. The rest of the PCR reaction mixture was composed of 1× Advantage 2 PCR buffer, 1× Advantage 2 polymerase mix (Clontech, Palo Alto, CA), 0.2 mM dNTP mix, 10% DMSO and 200 nM forward primer and reverse primer with a total reaction volume of 10 μl.

### RNA probe synthesis

From the cDNA sequence for each receptor we determined unique segments near the 5′ end to use as our *in situ* probes. They were generated using primers 1GSP13 and 1GSP8 for R-1, TKF1R2 and ABRev1bR2 for R-2, TKF1R3 and TK2R4R3 for R-3 (Table 1). The PCR products were gel purified as described in the QIAEX II Gel Purification Kit^©^ (QIAGEN) then inserted into the pGEM-T Easy vector (Promega, Madison, WI) and subsequently transformed into *E. coli* JM109 cells (Promega), per manufacturer's instructions. Overnight cultures were used for plasmid preparation with the Wizard Plus Miniprep system (Promega) or Fermentas GeneJET plasmid purification kit (Thermo Fisher Scientific, Inc., Waltham, MA), following the manufacturer's protocol. Purified plasmids were then sent for sequencing at the UNH's Hubbard Genome Center to verify the sequence and establish insert orientation.

The purified plasmids containing the lGnRH-R inserts were then digested overnight at 37°C separately with both SalI or NcoI restriction enzymes producing linearized templates for both sense and antisense probes. For R-3, SpeI was used in place of SalI given the presence of a SalI site in the 5′ end of the R-3 cDNA. Template DNA was purified using the QIAquick PCR purification kit (QIAGEN) per manufacturer's instructions. Riboprobes were synthesized using the SP6 or T7 Riboprobe Synthesis Kit (Promega) or the Maxiscript kit (Ambion, Carlsbad, CA), Digoxigenin (DIG)-labeled UTP (Roche, Indianapolis, IN) or fluorescein-12-UTP (Perkin Elmer Waltham, MA) as previously described (Root et al., 2005).

### *In situ* hybridization

The procedures for *in situ* hybridization followed those described by Root et al. (2005), Rubin et al. (1997), and Kavanaugh et al. (2008) with the following modifications: sections were cut at 18 μm at −20°C and there was no addition of RNase following hybridization. Sagittal tissue sections were cut the day of dissection on a cryostat (Reichert-Jung, Leica Instruments, Heidelberg, Germany). Sections were then mounted onto Superfrost Plus slides (VWR International, LLC Radnor, PA) and immediately moved into tissue fixing steps of the *in situ* hybridization protocol. Tissue sections were incubated with either anti-sense or sense riboprobes for lamprey GnRH-Rs. The application and signal-development of DIG-labeled or fluorescein-12-UTP probes followed previously described protocols (Rubin et al., 1997; Root et al., 2005; Kavanaugh et al., 2008). lGnRH-R-1, -2, and -3 were detected by the DIG colorimetric method. Additionally TSA kits for Cy3 and FITC (Perkin Elmer) were used to visualize R-2 and R-3. R-2 was detected by anti-DIG-HRP and TSA Cy3, R-3 by anti-fluorescein-HRP and TSA FITC.

Following signal development, DIG-labeled tissue sections were viewed under light microscopy using an Olympus BH2 microscope (Center Valley, PA), and digital photographs were captured with a QImaging Micropublisher 3.3 camera used with QCapture software (Surrey, BC, Canada). A detailed visual assessment was made of signal deposition for each slide. The Cy3, FITC labeled sections were analyzed using the confocal facilities at the UNH with a LSM 510 Meta laser-scanning confocal microscope (Zeiss, Thornton, NY) with visualization of the NBT-DF/BCI fluorescence by a 633-nm helium-neon laser as outlined by Trinh et al. (2007) using the following parameters: filter 638–799 nm, averaging 8, unmixed, pixel time 3.37 μs, 20× objective. Prolong Gold+DAPI, (Invitrogen) was used as the mounting media, which also allowed for the visualization of the stained nuclei via the mercury lamp.

## Results

### RT-PCR

**Figure 1:**
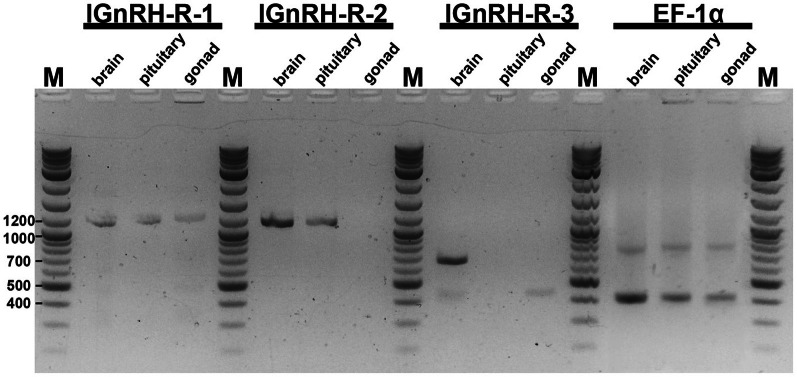
**Electrophoresis of RT-PCR products on a 1.8% TAE agarose gel.** The expected band size for each of the receptor RNA is as follows: lGnRH-R-1 at 1229 bp; lGnRH-R-2 at 1153 bp; and lGnRH-R-3 at 655 bp. EF-1α band size is 390 bp. In this figure, adult male brain, pituitary, and gonad tissues are shown. *M* = 2-Log ladder NEB (Beverly, MA); size (bps) of marker bands are indicated on the side.

Figure 1 is an example of the results obtained from the RT-PCR survey of the various lamprey tissues. The expected band size for each of the receptor RNA is as follows: lGnRH-R-1 at 1229 bp; lGnRH-R-2 at 1153 bp; and lGnRH-R-3 at 655 bp. The Elongation Factor 1α (house keeping) gene band size is 390 bp. In this figure, adult male brain, pituitary, and gonad tissues are examined. The presence of the 1229 bp band in all three tissues demonstrates expression of lGnRH-R-1 message. lGnRH-R-2 at 1153 bp band is present in the brain and pituitary while the expression of the message was not detected in the gonad. The 655 bp band of lGnRH-R-3 was only noted in the brain tissue. The EF-1α 390 bp band was present in all lanes with similar intensity. These results indicate that the cDNA synthesis was successful. Table 2 summarizes these results.

**Table 2:** **Results of receptor mRNA expression in various sea lamprey tissues by reverse transcriptase-PCR**.

	**Adult**						**Parasitic**						**Larval**		
	**Female**			**Male**			**Female**			**Male**					
lGnRH-R-	1	2	3	1	2	3	1	2	3	1	2	3	1	2	
Brain	+	+	+	+	+	+	+	+	+	+	+	+	+	−	−
Adeno-hypopysis	+	+	−	+	+	−	−	−	−	−	−	−	+	−	−
Gonad	−	−	−	+	−	−	−	−	+	−	+	+	−	#	+
Heart	#	#	+	#	+	+	−	+	−	−	+	−	−	−	−
Muscle	+	−	+	+	+	+	−	+	−	+	+	−	#	−	−
Liver	+	#	+	#	−	+	+	+	+	−	+	−	−	−	−
Eye	+	+	+	+	+	+	−	+	+	+	+	+	+	−	−
Intestine	+	+	+	+	+	−	+	+	−	−	+	−	−	−	−
Kidney	−	−	−	+	−	−	−	−	−	+	−	−	+	−	−
Skin	+	−	−	−	−	−	NA	NA	NA	NA	NA	NA	−	−	−
Thyroid	+	+	#	+	−	#	NA	NA	NA	NA	NA	NA	#	−	−
Gill	NA	NA	NA	NA	NA	NA	−	−	−	+	+	+	NA	NA	NA

Reactions were done on cDNAs made from pooled total RNA from individually extracted tissues in the adult and parasitic phase lampreys. In larval samples tissues were pooled first and then extracted. The following samples were pooled as follows: 4 per adults, 3 per parasitic phase lampreys, and 20 per larval lampreys.

+, message detected; −, message not detected; #, message detected only at 5× cDNA (100 ng/ reaction) concentration relative to original assay; NA, sample not available.

Table 2 shows the RNA expression of each of the three receptors in the various tissues. In the adult lamprey, lGnRH-R-1 was present in almost all tissues except the gonad and kidney of females and the skin of males. The RNA expression of lGnRH-R-2 and -R-3 is less widespread and varies slightly between each other. Also in adults, all three receptor RNA messages were present in both brain and eye tissues; however, lGnRH-R-3 RNA expression was not shown in either male or female pituitaries. lGnRH-R-1 was the only receptor shown in the adult male testes.

In parasitic phase lampreys, lGnRH-R-2 RNA expression was the most widespread in the various tissues (Table 2). All three receptors were detected in the brain. In gonadal tissue, lGnRH-R-3 was detected in both males and females, while lGnRH-R-2 was detected only in the testes. In the female, lGnRH-R-1 was not detected in the eyes. There was no detectable RNA expression of any of the three receptors in the pituitaries of either sex.

Tissues from larval lampreys had the lowest RNA expression (Table 2). lGnRH-R-1 RNA expression occurred in the brain, eye, pituitary, muscle, kidney, and endostyle. The only detected RNA expression of either lGnRH-R-2 or -3 was in the male and female gonads.

### *In situ* hybridization

**Figure 2:**
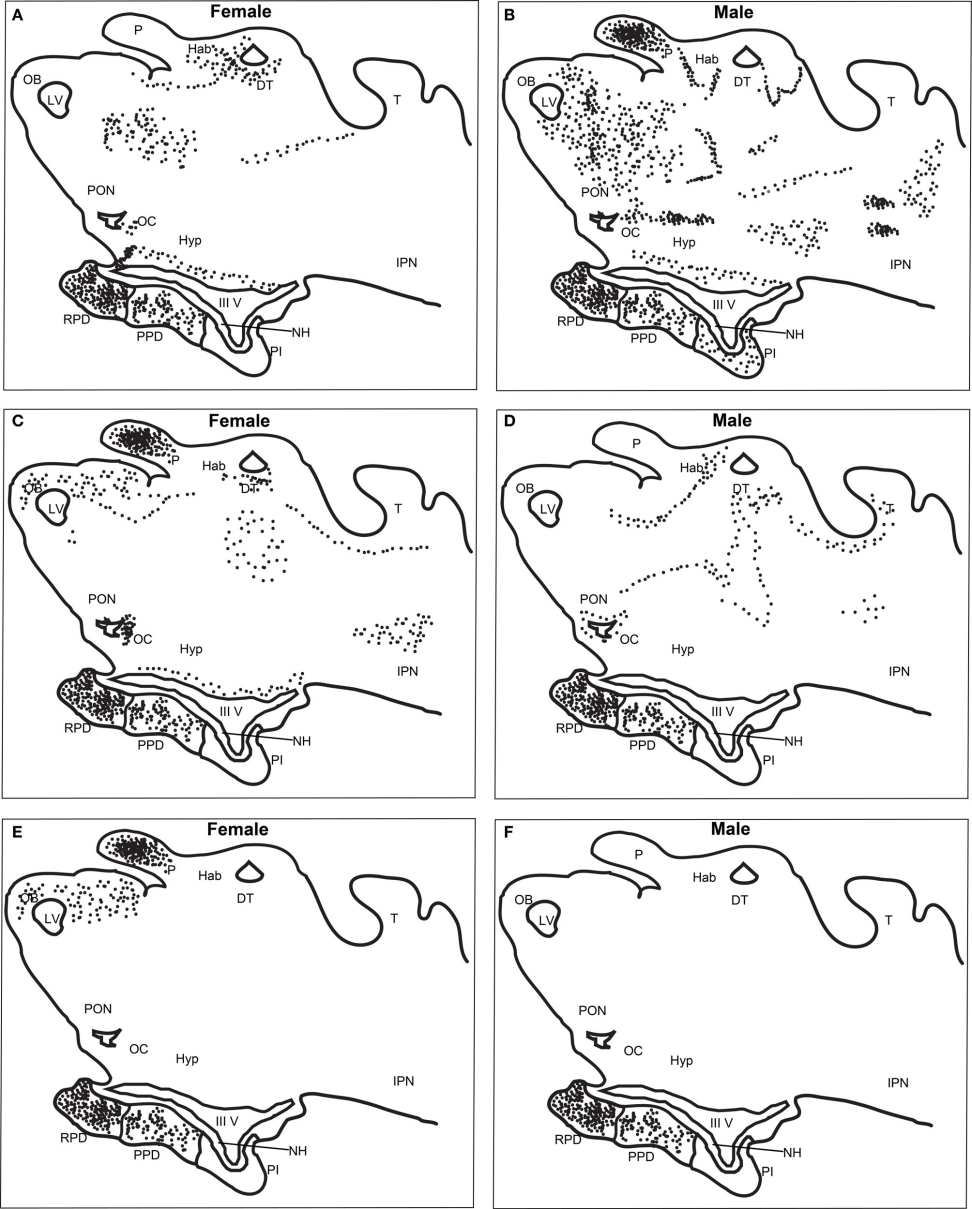
**Distribution of lGnRH-R-1, -2, and -3 RNA expression in adult female and male brains using *in situ* hybridization (DIG-labeled).** lGnRH-R-1 RNA expression shown in female **(A)** and male **(B)**, lGnRH-R-2 in female **(C)** and male **(D)**, and lGnRH-R-3 in female **(E)** and male **(F)**. Black dots are used to indicate the receptor message expression using the DIG system. Key for labeled features; DT, Dorsal Thalamus; Hab, Habenula; Hyp, Hypothalamus; IPN, Interpeduncular Nucleus; IIIV, Third Ventricle; LV, Left Ventricle; NH, Neurohypophysis; OB, Olfactory Bulb; OC, Optic Chiasm; P, Pineal; PON, Preoptic Nucleus; PI, Pars Intermedia; PPD, Proximal Pars Distalis; RPD, Rostral Pars Distalis; and T, Optic Tectum.

DIG-labeled, anti-sense lGnRH-R probes demonstrated moderate, yet distinct reaction product in specific neuronal nuclei across many regions of the adult lamprey brain, including the olfactory lobe, hypothalamus, habenular area, and midbrain (Figure 2). Slides were subjected to visual inspection under the microscope; major brain regions were scored for signal intensity on every slide. Patterns of staining were consistent between all brains examined and showed that the expression on the antisense slides was more intense than on the sense slides which had little or no staining. The data presented are representative of individual lampreys.

There was a noticeable difference of expression of lGnRH-R-1 between adult females and males in both the brain and the adenohypophysis (Table 3). The adenohypophysis of both females and males had intense staining in the rostral pars distalis (RPD) and moderate staining in the proximal pars distalis (PPD). However, only the males had light staining in the pars intermedia (PI). In the olfactory bulb (OB), females had light staining and the males had moderate staining. In the habenula, females showed intense expression, while males showed moderate expression. In contrast, only the pineal in males had intense staining. Also in males, the thalamus demonstrated intense staining, compared to the moderate staining in the females. There was moderate staining in the hypothalamus in the brains of both male and female lampreys. While in the adjacent anterior pre-optic area (POA), there was moderate staining in the males but staining was light in the female brains. In the mesencephalon, the males had intense staining and the females none.

**Table 3:** **Results of lGnRH Receptor-1, -2, and -3 RNA expression in the brain of female/male adult sea lamprey by *in situ* hybridization**.

**Regions of the brain and pituitary**	**lGnRH-R-1**		**lGnRH-R-2**		**lGnRH-R-3**	
	**Female**	**Male**	**Female**	**Male**	**Female**	**Male**
**TELENCEPHALON**						
Olfactory bulb	+	++	++	+	++	—
**DIENCEPHALON**						
Epithalamus						
Habenula	+++	++	+	+	—	—
Pineal	—	+++	+++	—	+++	—
Thalamus	++	+++	++	++	—	—
Hypothalamus	++	++	+	—	—	—
Anterior preoptic area	+	++	+	+	—	—
Mesencephalon	—	+++	++	++	—	—
**PITUITARY**						
Adenohypophysis						
Rostral pars distalis	+++	+++	+++	+++	+++	+++
Proximal pars distalis	++	++	++	++	++	++
Pars intermedia	—	+	—	—	—	—

Each column presents data representative of individual lampreys. +, light expression; ++, moderate expression; +++, intense expression; —, no detected expression.

lGnRH-R-2 showed a similar expression pattern in the adenohypophysis of adult males and females compared to lGnRH-R-1 except there was not any staining in the PI of the male pituitaries (Table 3). In the brain, the females displayed moderate staining in the OB. However, the males had light staining. Conversely to the expression of lGnRH-R-1, lGnRH-R-2 staining was absent in the male pineal and expressed intensely in the female pineal. The habenula was lightly stained in both males and females. In the thalamus, staining was again expressed moderately in female and males. In the hypothalamus, there was only light staining in the females. In males and females, there was light staining in the POA and moderate staining in the mesencephalon (Table 3).

Similarly, lGnRH-R-3 had intense staining in the RPD and moderate staining in the PPD of the pituitary in both adult males and females. In contrast to lGnRH-R-1 and R-2 in the males, there was no other staining for lGnRH-R-3 seen in the brain. In the females, the OB was lightly stained. The habenula showed no staining, but as with lGnRH-R-2 expression, the pineal in females was intensely stained. The other areas of the female brains did not display any staining (Table 3).

**Figure 3:**
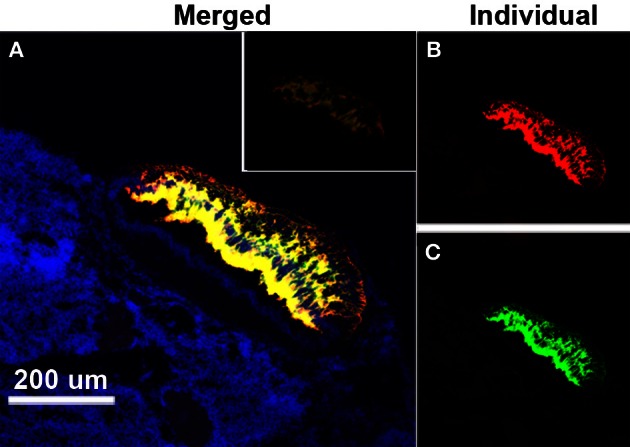
**Expression of lGnRH-R-2 and R-3 RNA in the pineal of a female lamprey using *in situ* hybridization. (A)** The merged channel (displayed as yellow) shows the composite of “anti-sense” expression of lGnRH-R-2 (Cy3-red channel) and lGnRH-R-3 (FITC-green channel) while DAPI (blue) is used to show the surrounding cell structure. The inset shows the control “sense” hybridization. The smaller panels have the lGnRH-R-2 **(B)** and lGnRH-R-3 **(C)** signals displayed individually.

The use of TSA fluorescent dyes gave the option of assaying for all three receptors simultaneously, but the best results were obtained when the hybridization only included lGnRH-R-2 (Cy3) and lGnRH-R-3 (FITC) (Figure 2). The results using the fluorescent probes were similar to those seen with the DIG labeled probes. It was seen that the staining in the pineal complex is strikingly limited to the pineal pellucida. While co-expression of lGnRH-R-2 and -3 showed extensive overlap in the pineal, lGnRH-R-2 was concentrated more dorsally while lGnRH-R-3 was concentrated in the mid-section of the pellucida (Figure 3).

## Discussion

Sea lampreys have three functional hypothalamic GnRHs (lGnRH-I, -II, and -III) and three functional pituitary GnRH receptors (lGnRH-R-1, -2 and -3) that are likely involved in mediating reproductive processes (Silver et al., 2005; Silver and Sower, 2006; Sower et al., 2009; Joseph et al., 2012). In our previous study in which the two novel forms of GnRH receptors were cloned, lGnRH-R-2 precursor transcript was detected in a wide variety of tissues including the pituitary whereas lGnRH-R-3 precursor transcript was not as widely expressed and primarily expressed in the brains and eyes of adult male and female lampreys (Joseph et al., 2012). In the current study, the RNA expression of lamprey GnRH receptors were more widely expressed in adult tissues as compared to parasitic-phase and least widely expressed in the larval tissues. There were noted differences in male and female lampreys in the adult and parasitic phases for all three receptors. The data showed the presence of all three receptor transcripts in brain tissues for adult and parasitic phases and all three receptor transcripts were expressed in the adult pituitaries (adenohypophysis), but not in the parasitic pituitaries. However, in the larval phase, only lGnRH-R-1 is expressed in the larval brain and pituitary. *In situ* hybridization revealed that lGnRH-R-2 and -3 were only expressed in the pineal tissue of female adult lampreys compared to males, restricted to the pineal pellucida. The results of these studies suggest differential regulation within males and females at different reproductive stages.

In comparison to the GnRH ligands, the GnRH receptors are much more widely expressed in the adult brain of the sea lamprey. The adult stage is when the sea lampreys have returned to freshwater streams and undergo the final maturational processes resulting in mature eggs and sperm, spawning and then death (Fahien and Sower, 1990; Bolduc and Sower, 1992; Sower et al., 2011). Unlike most other vertebrates, the general pattern of distribution of lamprey GnRH-I, -II and -III neurons are restricted and localized to the anterior-preoptic-neurohypophysial tract as determined by immunocytochemical studies (Nozaki and Gorbman, 1984; King et al., 1988; Nozaki et al., 2000; Kavanaugh et al., 2008). In the preoptic area and anterior pituitary, there was a general similarity of distribution pattern of the lGnRH and the lGnRH receptors, with the exception of lGnRH-R-3 in the males. This is similar to that noted for other vertebrates in which the distribution of GnRH receptor expression corresponds to the projection of GnRH ligands (Chen and Fernald, 2008). However, the GnRH receptors in the adult lamprey brain were more widely expressed in the thalamus and pineal, as well as the olfactory lobe, and do not correspond to the distribution of the GnRH ligands.

Using *in situ* hybridization, we showed that lGnRH-R-1, -2, and -3 are present in the PPD and RPD of the pituitary. In our earlier study and current study, using RT-PCR, we only showed lGnRH-R-1 and -2 being present in the pituitary. This discrepancy may reflect the low expression of lGnRH-R-3 that may be difficult to detect by RT-PCR using poly-adenylated RNA as a source of the cDNA and not gene-specific amplification. lGnRH-R-1 is the only receptor in lampreys in which lGnRH-I can bind and transduce both IP and cAMP signal transduction (Silver and Sower, 2006). However, each of the three lamprey GnRH ligands can stimulate each of the three receptors via IP signal transduction with varying binding affinity and potency (Joseph et al., 2012). lGnRH-R-2 has a higher binding affinity and IP potency in response to lGnRH-III than lGnRH-II, whereas lGnRH-R-3 has a higher binding affinity and IP potency in response to lGnRH-II than lGnRH-III (Joseph et al., 2012). Although *in vitro* ligand selectivity does not necessarily correlate with *in vivo* functional significance (Grosse et al., 2000), it is likely that lGnRH-I, -II, and -III are all physiological regulators of the pituitary gland in the sea lamprey. This is supported by various studies examining the changes of GnRH ligands during this final maturational period.

However, it is not known at this time how the three GnRHs regulate and control the pituitary glycoprotein hormone (gonadotropin) (Sower et al., 2009). The distribution of the GnRH receptors in the RPD in the current study is unexplained at this time. Unlike all jawed vertebrates, only one pituitary glycoprotein hormone has been identified in the agnathans, lamprey and the hagfish (Sower et al., 2006; Uchida et al., 2010). Our studies in lampreys suggest the involvement of one pituitary glycoprotein hormone and two glycoprotein hormone receptors as opposed to three or four dimeric hormones and three receptors in gnathostomes (jawed vertebrates) (Freamat et al., 2006; Freamat and Sower, 2008). The immunohistochemical data of the adult lamprey pituitary only show the PPD cells that produce gonadotropin (GTH); while adrenocorticotropin hormone (ACTH) cells are produced in the RPD; growth hormone (GH) and GTH cells are produced in the PPD; and melanocyte stimulating hormone (MSH) cells are in the PI (Nozaki et al., 1995; Sower et al., 2006). When we first identified the beta subunit of GTH in lamprey, we tested the biological activity of GnRH-I and -III on the pituitary sea lamprey hormones. The results demonstrated that lamprey GnRH not only stimulated GTH but also stimulated expression of GH, but not of proopiocortin (POC) and proopiomelanotropin (POM) (Sower et al., 2006). Once the structure(s) of the lamprey pituitary glycoprotein hormone(s) are fully determined, then the interrelationships between each of the three GnRH receptors and the glycoprotein hormone(s) can be assessed.

More recently, changes in lGnRH-I, -II, and -III, ovarian morphology and plasma estradiol were examined during the final two months of the reproductive season of adult male and female sea lampreys (Sower et al., 2011). The results from this study showed significant correlations between water temperature, fluctuation of brain GnRHs, plasma estradiol, and reproductive stages during this time. In males, lGnRH-I concentration increased early in the season, peaked, then declined with a subsequent increase with the final maturational stages. In comparison, lGnRH-II and -III concentrations were also elevated early in the season in males, dropped, and then peaked in mid-season with a subsequent decline of lGnRH-II or increase of lGnRH-III at spermiation. In females, lGnRH-III concentration peaked in mid-season with a drop at ovulation while lGnRH-I remained unchanged during the season. In contrast, lGnRH-II concentrations in females were elevated at the beginning of the season and then dropped and remained low during the rest of the season. The dynamics of the binding of the three lamprey GnRH receptors have not been examined through this final maturation period. However, prior to the identification of the lamprey GnRH receptors, two high affinity-binding sites were characterized in the adult lamprey pituitary (Knox et al., 1994). Subsequent studies showed that the concentration of these two high affinity-binding sites increased during the final maturational period peaking near and at ovulation (Materne et al., 1997). Further studies will be needed to understand the dynamics and interactions of the three GnRH ligands with each of the three GnRH receptors in adult male and female lampreys.

As reviewed in Falcon et al. (2010), some previous studies have shown that the pineal organ and melatonin are connected and/or involved in the brain and the daily and seasonal reproductive patterns in fish. In the dogfish, *Scyliorhinus canicula*, a fluorescent carbocyanine (DiI) was applied to the pineal organ and brain showing an extensive bilateral projection (Mandado et al., 2001). These authors compared these results with GnRH-immunocytochemical analyses of the dogfish brain and suggested that there were pineal projections to the midbrain GnRH-immunoreactive neurons. In the European sea bass, *Dicentrarchus labrax*, the pineal organ was shown to receive GnRH-2 immunoreactive fibers originating from the synencephalic neurons by immunohistochemistry and shown to express a GnRH receptor by *in situ* hybridization (Servili et al., 2010). Using physiological experiments, these authors further demonstrated that GnRH-2 could stimulate melatonin release. In the current study, *in situ* hybridization showed that lGnRH-R-2 and -3 were expressed in the pineal tissue of adult lampreys, restricted to the pineal pellucida. In lampreys, as in some other vertebrates, there are pineal extensions to various areas in the diencephalon as well as the mid-brain (Yanez et al., 1993; Mandado et al., 2001); although there are no pineal fibers to the preoptic region-anterior hypothalamus in the silver lamprey (Puzdrowski and Northcutt, 1989). The significance of the presence of two forms of lGnRH receptors in the pineal of female lampreys, restricted to the pineal pellucida, and its relationship to melatonin and reproduction is intriguing and remains to be determined.

In general, the system of GnRH neurons is intimately connected to the olfactory system from early development and functionally into adulthood in vertebrates that have been studied (Tobet et al., 1996). The exception appears to be adult lampreys. In the adult lampreys, all three GnRH receptors were expressed in the OB. However, there is a general absence of GnRH cells and fibers in the olfactory system in adult lamprey brains (Nozaki and Gorbman, 1984; King et al., 1988; Kavanaugh et al., 2008) suggesting that olfactory stimuli may not play a major role in GnRH secretion in adult lampreys. In larval lampreys, there are a few ir-GnRH-III fibers that are visible in the olfactory regions that originate from the more caudal cells found in the preoptic area/hypothalamus (Tobet et al., 1995). In larval lampreys, both lamprey GnRH-I and -III are found in the cell bodies in the rostral hypothalamus and preoptic area (Wright et al., 1994; Tobet et al., 1995). However, in the larval phase, only lGnRH-R-1 is expressed in the larval brain and pituitary. Interestingly, lGnRH-R-I has the highest selectivity to ligand lGnRH-III, which happens to be the predominant immunoreactive form in larval lampreys (Tobet et al., 1995). In the parasitic phase of the sea lamprey, the distribution of GnRH has not been studied. However, it has been shown that the period of metamorphosis from larval to parasitic state is a prominent phase of up-regulated activity for hypothalamic lGnRH-I and -III corresponding to gonadal growth (Youson et al., 2006). It was postulated that based on the overlap of olfactory- and GnRH-containing fibers from prolarval stages to metamorphosis, olfactory stimuli may play a major role in the regulation of GnRH secretion in lampreys (Tobet et al., 1995). Further studies are warranted to examine the relationship of the lGnRH ligands and receptors in male and females during the larval to parasitic stage.

In conclusion, the current study identifies the distribution of three GnRH receptors, particularly in female and male lamprey brains and pituitaries using both RT-PCR and *in situ* hybridization. Differential expression of each of the three receptors in the brain and pituitary of male and female lampreys at three different life/reproductive stages provides an initial comparative analysis of expression. In addition, it provides a means for further investigation of the complexity of the GnRH-GnRH receptor system along with the interactions of other neurohormone systems.

### Conflict of interest statement

The authors declare that the research was conducted in the absence of any commercial or financial relationships that could be construed as a potential conflict of interest.
